# Targeted O-glycoproteomics for the development of diagnostic markers for advanced colorectal cancer

**DOI:** 10.3389/fonc.2023.1104936

**Published:** 2023-02-09

**Authors:** Daisuke Takakura, Shoko Ohashi, Noritoshi Kobayashi, Motohiko Tokuhisa, Yasushi Ichikawa, Nana Kawasaki

**Affiliations:** ^1^ Graduate School of Medical Life Science, Yokohama City University, Yokohama, Japan; ^2^ Department of Oncology, Yokohama City University Hospital, Yokohama, Japan

**Keywords:** glycoproteins, glycosylation, O-glycoproteomics, serum diagnostic marker, colorectal cancer, LC/MS/MS

## Abstract

Aberrant glycosylation is a prominent feature of cancer, that can be used as targets to improve the existing cancer biomarkers, and help to assess metastasis risks, and therapeutic effects. We developed a targeted O-glycoproteomics method using serum specimens, and evaluated its utility in identifying advanced colorectal cancer (CRC) markers. To this end, we combined consecutive lectin affinity purification using *Maclura pomifera* lectin (MPL), jacalin, and *Sambucus nigra* lectin, which have affinities for the following O-glycans, that have received attention as cancer-related antigens, Tn (GalNAc-Ser/Thr), Sialyl Tn (Siaα2-6GalNAc-Ser/Thr), T (Galβ1-3GalNAc-Ser/Thr), Sialyl T (Siaα2-3Galβ1-GalNAc-Ser/Thr), and di-Sialyl T (Siaα2-3Galβ1-3[Siaα2-6] GalNAc-Ser/Thr), with a unique O-glycoproteomics approach. A total of 2,068 O-glycoforms derived from 265 proteins were identified in healthy individuals and patients with advanced CRC, of which 44 CRC-specific O-glycoforms were extracted. Particularly, five glycoproteins with T, Sialyl T, and di-Sialyl T antigens in specific peptide regions were evaluated quantitatively and statistically. We found that fibulin-2 (FBLN2) (aa330-349)/T antigen (area under the curve [AUC] = 0.92); macrophage colony-stimulating factor 1 (CSF1) (aa370-395)/(T + di-Sialyl T) (AUC = 0.94); macrophage mannose receptor 1 (MRC1) (aa1083-1101 and aa1215-1229)/T (AUC = 0.96 and 0.99); fibrinogen alpha chain (FGA) (aa354-367, aa511-527 and aa559-573)/Sialyl T (AUC = 0.98, 0.90 and 0.94); and complement component C7 (C7) (aa692-701)/di-Sialyl T (AUC = 1.00), can have high diagnostic efficacy to strategically predict advanced CRC groups. Hence, they could be promising markers for detection of advanced CRC, and provide new clinical test indicators along with lectins, such as MPL and jacalin. Our O-glycoproteomics platform provides a novel tool and resource, for researchers and clinicians seeking to better understand and treat advanced CRC.

## 1 Introduction

Advanced colorectal cancer (CRC) is associated with mental and physical burden on patients. The recent increase in aging population, and the growing incidence of colorectal cancer at all ages have caused more than half a million deaths worldwide (1). The prognosis of advanced CRC is highly heterogeneous, leading to diverse clinical outcomes, that are associated with complex molecular mechanisms (2). A noninvasive fecal occult blood test followed by colonoscopy is recommended for the clinical diagnosis of advanced CRC (3). In addition, genetic testing is required to help determine the treatment strategies for advanced CRC (4). However, for these clinical tests to be recognized as viable tools, all asymptomatic people should be provided with more favorable evidence of diagnostic certainty, and cost-effectiveness. Therefore, various omics approaches, such as genomics (5, 6), metabolomics (7, 8), and proteomics (9, 10), have been used to explore novel clinical test indicators to enable identification of patients at high risk of metastasis, and selection of effective treatment options best suited to the patient’s condition.

Protein glycosylation is the predominant posttranslational modification in mammalian cells (11). Glycans of glycoproteins are classified into two types: N-glycans, which are linked to the Asn residue in the tripeptide sequence (Asn-X-Ser/Thr, X≠Pro), and O-glycans, linked to Ser and Thr residues (12). Mucin-type (O-GalNAc type) O-glycans begin with an *N*-acetylgalactosamine (GalNAc) α-linked to the hydroxyl group of Ser and Thr residues. This initial step is catalyzed by 20 different *N*-acetylgalactosaminyltransferases (GalNAc transferases; GALNTs) (13), followed by differential expression of various other transferases to synthesize complex O-glycans. Over 85% of all proteins with a signal sequence in the secretory pathway are modified with mucin-type O-glycans to regulate, or fine-tune the functions of specific proteins (14–16).

Aberrant glycosylations have been used as diagnostic marker in cancer. Highly glycosylated carcinoembryonic antigen (CEA), and carbohydrate antigen 19-9 (CA19-9; sialyl Lewis A) are commonly used clinical test indicators, before and after surgery, in several cancer, including CRC. However, their diagnostic reliability is limited by poor sensitivity and specificity (17). For instance, the detection sensitivity of CEA was 64.5, and the area under the curve (AUC) value for diagnostic ability evaluation was 0.79 for the diagnosis of CRC (18). Therefore, in search for more reliable markers other than CEA and CA19-9, many researchers have focused on aberrant N-glycoproteins in cancer, and have evaluated their clinical usefulness as markers; the representative of which is alphafetoprotein-L3 (AFP-L3), as a marker for hepatocellular carcinoma. Core-fucosylated αAFP, which is characteristically present in the serum of patients with hepatocellular carcinoma, is detected by combining the core fucose recognition lectin (*Lens culinaris* agglutinin; LCA), with an anti-αAFP antibody (19–21). In addition, core-fucosylated haptoglobins are promising diagnostic marker candidates for pancreatic cancer (22, 23).

Abnormal mucin-type O-glycans are frequently observed in various cancers. In particular, neosynthesis and over-modification of simple mucin-type O-glycans, such as the Tn antigen (GalNAc-Ser/Thr), Sialyl Tn antigen (Siaα2-6GalNAc-Ser/Thr; STn), and T antigen (Galβ1-3GalNAc-Ser/Thr), play important roles in tumor progression and malignancy in various cancers, and are associated with changes in protein conformation and signaling (24–30). These typical mucin-type O-glycans with limited structural heterogeneity changes in relation to disease and metabolic status can be used for diagnostic and therapeutic interventions (31). However, compared to N-glycoproteomics, O-glycoproteomics is much more challenging because of the absence of efficient enrichment methods for intact O-glycopeptides. There remains a concern regarding the efficiency of recovery of typical mucin-type O-glycopeptides with representative N-glycopeptide enrichment methods like hydrophilic interaction chromatography (32–34) or acetone (35–37). The valuable method for concentrating mucin-type O-glycopeptides using GlycOCATCH affinity resin is limited to asialoglycopeptides, because the presence of sialic acid inhibits the affinity for the resin (38). The efficient enrichment methods for the vast majority of intact mucin-type O-glycopeptides are thus awaited in the field of diagnostic research.

In this study, we developed an O-glycoproteomic platform to search for advanced CRC diagnostic markers based on changes in glycosylation of serum proteins with typical mucin-type O-glycans. The platform consisted of lectin affinity fractionation, glycopeptide enrichment, and liquid chromatography with tandem mass spectrometry (LC-MS/MS) with two normalized collision energy (NCE) parameters. Since O-glycoproteins carrying Tn, STn, T, Sialyl T (Siaα2-3Galβ1-GalNAc-Ser/Thr; ST), and di-Sialyl T (Siaα2-3Galβ1-3[Siaα2-6] GalNAc-Ser/Thr; di-ST) antigens, which have received attention as cancer-related antigens were targeted as diagnostic markers for advanced CRC, *Maclura pomifera* lectin (MPL), jacalin, and *Sambucus nigra* lectin (SNL), which have affinity for these antigens, were used for consecutive lectin affinity purification. These lectins can also be used for the detection of marker O-glycoproteins. Acetone-based glycopeptide enrichment, targeting sialoglycopeptides, was combined with the purification method using GlycOCATCH affinity resin for asialoglycopeptides to achieve highly efficient enrichment of the vast majority of intact mucin-type glycopeptides. The identified proteins were evaluated for their potential to be used as diagnostic markers in serum from patients with advanced CRC.

## 2 Materials and methods

### 2.1 General information

All chemicals used were of guaranteed reagent and mass spectrometry grade, and were purchased from Promega (WI, USA), FUJIFILM Wako Pure Chemical (Osaka, Japan), Kanto Chemical (Tokyo, Japan). Magnetic beads modified with streptavidin (SA) were purchased from Vector Laboratories (Burlingame, UK). The biotin-modified MPL, jacalin, and SNL were obtained from Vector Laboratories.

### 2.2 Clinical specimens and ethics

Human serum samples were collected from Yokohama City University Hospital in Yokohama, Japan. Serum samples were obtained from 8 patients, and 10 healthy donors who provided informed consent, and the study was approved (No. B160301015) by the Institutional Ethics Committee of Yokohama City University (Kanagawa, Japan). All cancer diagnoses were based on the histopathological methods. All samples were anonymized, and the gender, age, cancer-related laboratory pathological results, and pathological diagnoses were recorded. Clinical characteristics of advanced CRC patients are shown in **Table 1**.

**Table 1:** Clinical characteristics of advanced CRC samples.

Variable	Advanced CRC (n = 8)	Control (n = 10)
Sex		
Male	5	5
Female	3	5
Age		
Mean	69.4	38.3
Range	62–78	24–60
Pathology		
Tubular Ad	2	
Well Differentiated Ad	4	
Moderately Differentiated Ad	2	
TNM stage		
IIc	1	
IV	7	
Tumor Marker (CEA/CA19-9)		
Positive (+/+)	3	
Negative (−/−)	5	

CRC, colorectal cancer; Ad, adenocarcinoma; TNM, tumor, node and metastasis; CEA, carcinoembryonic antigen; CA19-9, carbohydrate antigen.

### 2.3 Purification of O-glycoprotein by lectin affinity

All serum proteins were quantified using the bicinchoninic acid method, and 80 μg of serum protein was enriched with MPL. Biotinylated MPL (5 μL; 10 μg) was diluted with 195 μL Tris-buffered saline (TBS) with 0.1% Triton X-100 (TBS-Tx). It was then added to SA magnetic beads that were repeatedly washed with TBS-Tx, and incubated for 30 min at 4°C in a thermomixer (Eppendorf, Hamburg, Germany) under continuous shaking at 1,400 rpm. After washing three times with TBS-Tx, the pre-cleared serum protein was added to the MPL-bound beads and incubated for 16 h at 4°C under the same shaking conditions. The MPL-bound proteins were thoroughly washed with TBS-Tx, and recovered by heating in TBS containing 0.2% sodium dodecyl sulfate at 95°C. The MPL-unbound proteins (supernatant) were recovered in a new collection tube and subjected to jacalin purification. The second affinity purification step, with biotinylated jacalin (2.6 μL; 13 μg), was performed using the same procedure as that used for purification with MPL. The third affinity purification step, with SNL (2.5 μL; 7 μg), was performed using the method described above. All lectin-bound proteins were combined in the same collection tube.

### 2.4 Trypsin/Lys-C digestion and glycopeptide enrichment

Intact O-glycopeptides were prepared by a combination of enrichment methods using acetone (35) and GlycOCATCH (Genovis, Lund, Sweden) enrichment resin for affinity purification of mucin-type O-glycopeptides. O-glycoproteins that were consecutively affinity-purified with the three types of lectins as described in section 2.3, were precipitated by adding a three-fold volume of cold acetone and incubating for 16 h at -30°C, followed by centrifugation at 12,000 × *g* for 10 min at 4°C. The precipitate was reduced with 10 mM dithiothreitol at 56°C for 30 min, and alkylated with 20 mM iodoacetamide at 25°C in dark for 40 min. Subsequently, O-glycoproteins were digested with 1.8 µg of trypsin/Lys-C mix (Promega, WI, USA) for 16 h at 37°C, in a thermomixer under continuous shaking at 800 rpm. O-glycopeptides were precipitated with a five-fold volume of cold acetone, incubated for 16 h at -30°C, and then centrifuged at 12,000 × *g* for 10 min. The supernatant was collected in a new tube, dried using a speed vacuum concentrator, and subjected to GlycOCATCH affinity purification. O-Glycopeptide enrichment was performed according to the manufacturer’s instructions. The resin was washed three times with TBS, and the supernatant fraction of acetone reconstituted O-glycopeptides in TBS-Tx, was rotated with a resin containing 10 units of sialidase mix (SialEXO, Genovis) at 25°C for 2 h. Sialidase mix catalyzes the hydrolysis of α2-3, α2-6, and α2-8 linked sialic acid residues from O-glycopeptide. This enzymatic treatment is required for complete removal of sialic acids, the presence of which reduce the affinity of the O-glycopeptides for the resin. The unbound peptides were removed by centrifugation at 1,000 × *g* for 1 min and washed three times with TBS containing 0.5 M NaCl. Resin-bound peptides were recovered by shaking with 8 M urea for 5 min. It was combined with the glycopeptide precipitate using the acetone enrichment method and purified with GL-Tip SDB (GL Science, Tokyo Japan).

### 2.5 LC/MS/MS and label-free quantification of O-glycoforms

Peptides and glycopeptides were separated on an EASY-nLC 1000 (Thermo Fisher Scientific, MA, USA), with an Acclaim PepMap100 C18 LC column (75 µm × 20 mm, 3 µm, Thermo Fisher Scientific), and a nano HPLC capillary column (75 µm × 120 mm, 3 µm, C18; Nikkyo Technos, Tokyo, Japan). The mobile phase consisted of water containing 0.1% v/v formic acid (pump A) and acetonitrile containing 0.1% v/v formic acid (pump B). The peptides and glycopeptides were eluted at a flow rate of 0.3 µL/min with a linear gradient from 0 to 35% B over 120 min. Mass spectra were acquired on a Q Exactive mass spectrometer (Thermo Fisher Scientific), equipped with a Nanospray Flex Ion Source (Thermo Fisher Scientific) operated in positive ion mode. We used an Xcalibur 4.4 workstation (Thermo Fisher Scientific) for MS control and data acquisition. The spray voltage was set at 1.8 kV, the capillary temperature was maintained at 250°C, and the S-lens (stacked ring ion guide) RF (radio frequency) level was 50. Full mass spectra were acquired using an *m/z* range of 350–2000 with a resolution of 70,000. The product ion mass spectra were acquired against the 20 most intense ions using a data-dependent acquisition method with a resolution of 17,500 with normalized collision energy (NCE) of 27 and 35, and exclusion of 30s. In general, the required NCE differs depending on the size of O-glycopeptide and the composition of O-glycan. The average peak area values traced for the precursor ion of O-glycoform from two replicates using different NCEs were calculated for all serum samples.

Raw data files were processed using the Byonic search engine ver. 3.11 (Protein Metrics, CA, USA), integrated as a node into Proteome Discoverer ver. 2.4 (Thermo Fisher Scientific). The UniprotKB database for humans (status/2022/02), and in-house O-glycan database (10 entries; HexNAc(1), HexNAc(2), HexNAc(1)Hex(1), HexNAc(1)Hex(1)Fuc(1), HexNAc(2)Hex(1), HexNAc(1)Hex(1)NeuAc(1), HexNAc(1)Hex(2)Fuc(1), HexNAc(1)Hex(1)Fuc(1)NeuAc(1), HexNAc(1)Hex(1)NeuAc(2), and HexNAc(1)NeuAc(1)) were used for the database search. The search conditions were as follows: trypsin: maximum number of missed cleavages, 2; static modification: carbamidomethylation (C); dynamic modifications: Gln > PyroGlu (N-term Q), oxidation (M); precursor mass tolerance: 10 ppm; fragment mass tolerance: 20 ppm; and maximum number of O-glycosylations per peptide: 2. Following the search, all identified peptides were filtered at a false discovery rate (FDR) threshold of 1% using a target/decoy search strategy. The identified peptides were evaluated for reliability using the percolator node, and only the high-confidence O-glycoforms were processed using Microsoft Excel ver. 2209 (Microsoft, WA, USA). Of the O-glycoforms identified by the Byonic search engine, the product ion spectra of notable O-glycoforms were manually inspected and validated. Manual annotation of the relevant oxonium ions was performed to confirm the monosaccharide composition of the attached O-glycans. The typical oxonium ions were annotated on the spectra, including *m/z* at 204.08 (HexNAc), 274.09 and 292.10 (NeuAc), and 366.14 (HexNAc + Hex). The peptide and O-glycan masses of the O-glycoforms were deduced from the molecular masses of the peptide ion, which is commonly found to be more intense.

Label-free quantification of O-glycoforms was performed using a combination of Minora Feature Detector, Feature Mapper, and Precursor Ions Quantifier nodes in Proteome Discoverer 2.4 (Thermo Fisher Scientific). The analytical conditions were as follows: Peptide to Use, unique; Precursor Quantification, area; and Normalization mode, none. Measured changes of O-glycoform were not normalized to changes in total peptide abundance, because of the bias caused by enrichment based on lectin affinity which has important implications.

### 2.6 Statistical data analyses

Principal component analysis (PCA), and Wilcoxon matched pairs signed rank test were used to compare the glycoform peak areas between CRC, and normal serum samples. *P*-values were adjusted using the Benjamini-Hochberg method for controlling the 1% false discovery rate (FDR). The differences with *p* < 0.01 were considered significant. Volcano plots were prepared to present increase in O-glycoforms in CRC patient sera. The log2 fold change for O-glycoforms were plotted against the −log10 of the *p*-value. Statistical significance was declared at FDR LogWorth (−log10 *p*-value) of 1.3 (equivalent of a *p*-value of 0.05). The diagnostic performance of O-glycoprotein was evaluated using area under the receiver operating characteristic (ROC) curves. Statistical analyses were performed using the JMP Pro 15 software (SAS Institute, NC, USA).

## 3 Results

### 3.1 Lectin affinity purification and targeted O-glycoproteomics

**Figure 1:**
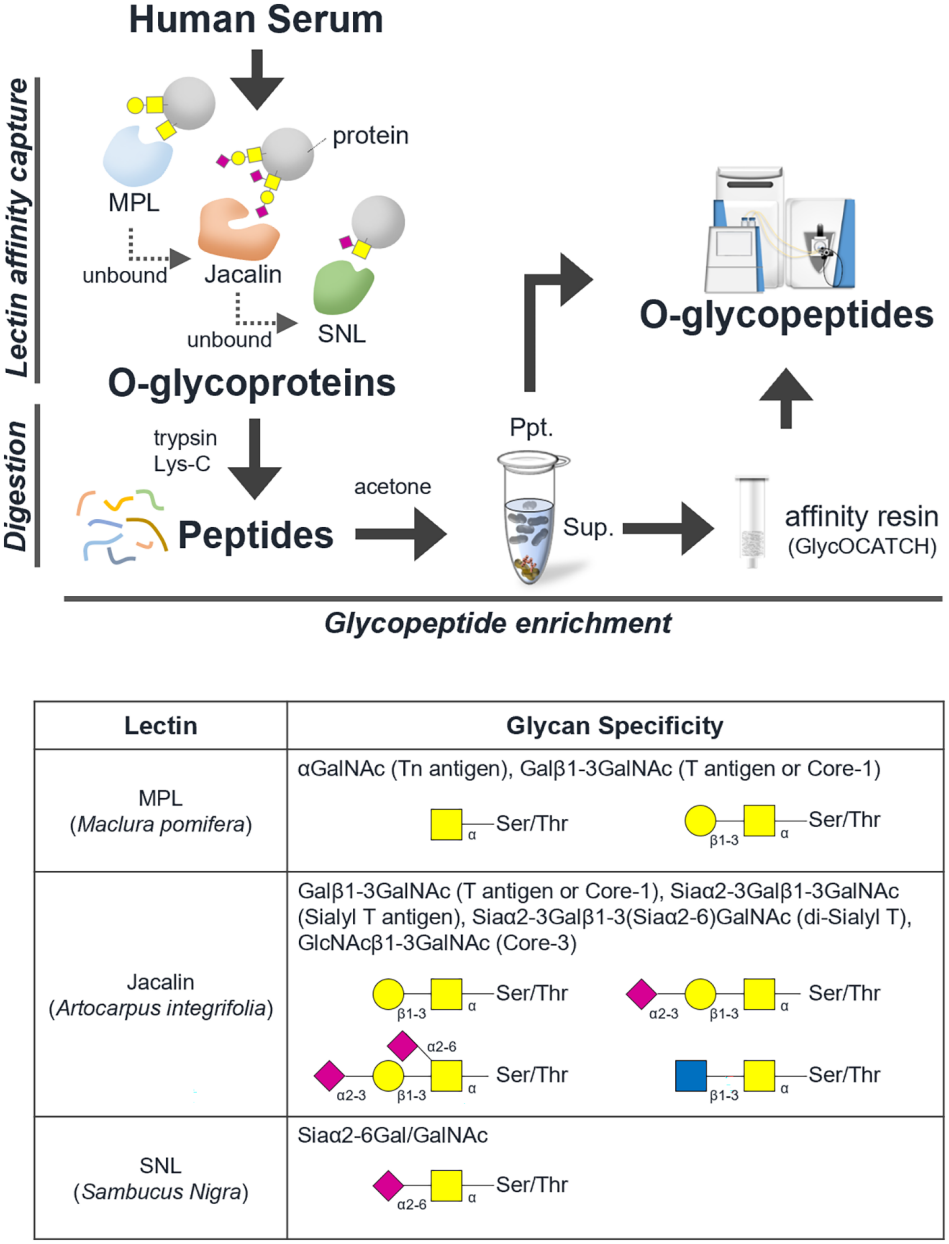
Schematic workflow of O-glycoproteomics. The comprehensive analysis of intact O-type glycopeptides was broadly divided into three stages. Step 1: Concentration of O-glycoprotein by lectin affinity, Step 2: trypsin/lysyl endopeptidase digestion, Step 3: concentration of O-glycopeptide using acetone and GlycOCATCH affinity resin. A list of glycan specificities of lectins used in this study is shown.

**Figure 2:**
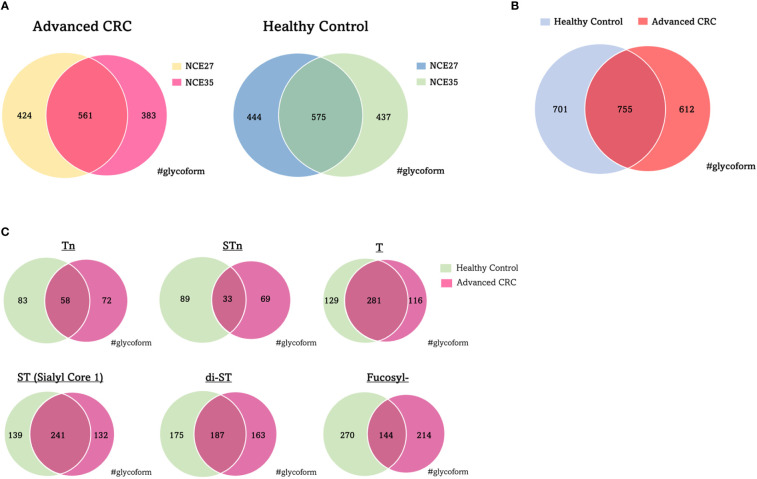
Qualitative comparison of O-glycoforms identified by LC/MS/MS. **(A)** Venn diagrams of O-glycoforms with different NCE. **(B)** Venn diagram of O-glycoforms identified in serum with advanced CRC patients and healthy individuals. **(C)** Venn diagram showing the distribution of typical O-glycoforms identified in serum with advanced CRC patients and healthy individuals. NCE, normalized collision energy; CRC, colorectal cancer.

**Figure 1** shows a schematic workflow of label-free quantitative targeted O-glycoproteomics for detection of Tn, STn, ST, T, and di-ST antigen-bound proteins in serum. Comprehensive analysis of O-glycopeptides can be broadly divided into three stages. First, target O-glycoproteins were concentrated based on lectin affinity. MPL was used to enrich Tn and T antigen-bound proteins in the serum of patients with advanced CRC and healthy individuals. In the MPL-unbound fraction, ST, and di-ST-bound proteins were enriched by jacalin. STn and Sialyl core 1 (Galβ1-3[Siaα2-6] GalNAc)-bound proteins were recovered from the jacalin-unbound fraction by SNL. Second, enriched O-glycoproteins were digested with trypsin and Lys-C. Third, O-glycopeptides were enriched using acetone and GlycOCATCH affinity resin. Most of the sialylated glycopeptides, such as STn, ST, Sialyl core 1, and di-ST, and some of the other glycopeptides were collected *via* acetone precipitation, as previously reported (34). The remaining glycopeptides were recovered from the supernatant using GlycOCATCH affinity resin, which captures T antigen-bound peptides. The glycopeptides were subjected to label-free quantification using LC-MS/MS with the NCE set to 27 or 35, and the resulting spectral data were processed with the Byonic search engine. The MS raw data files were deposited in the ProteomeXchange Consortium *via* the jPOST partner repository (http://jpostdb.org) with the dataset identifiers PXD035279 and PXD035280. **Figure 2** shows a qualitative comparison of the O-glycoforms identified using LC-MS/MS. LC-MS/MS with NCE of 27 and 35 resulted in 1,367 and 1,456 O-glycoforms in the serum of patients with advanced CRC and healthy individuals, respectively (**Figure 2A**). Overall, 2,068 O-glycoforms, derived from 265 proteins, were identified. Among these, 612 O-glycoforms were found in patients with advanced CRC, 701 in healthy individuals, and 755 O-glycoforms were common in both groups (**Figure 2B**). T antigen and ST/Sialyl Core 1 were commonly found in the serum of patients with advanced CRC and healthy individuals. The Venn diagrams highlight the differences in typical O-glycoforms identified in the serum of patients with advanced CRC, and healthy individuals (**Figure 2C**).

### 3.2 CRC-related O-glycoproteome

**Figure 3:**
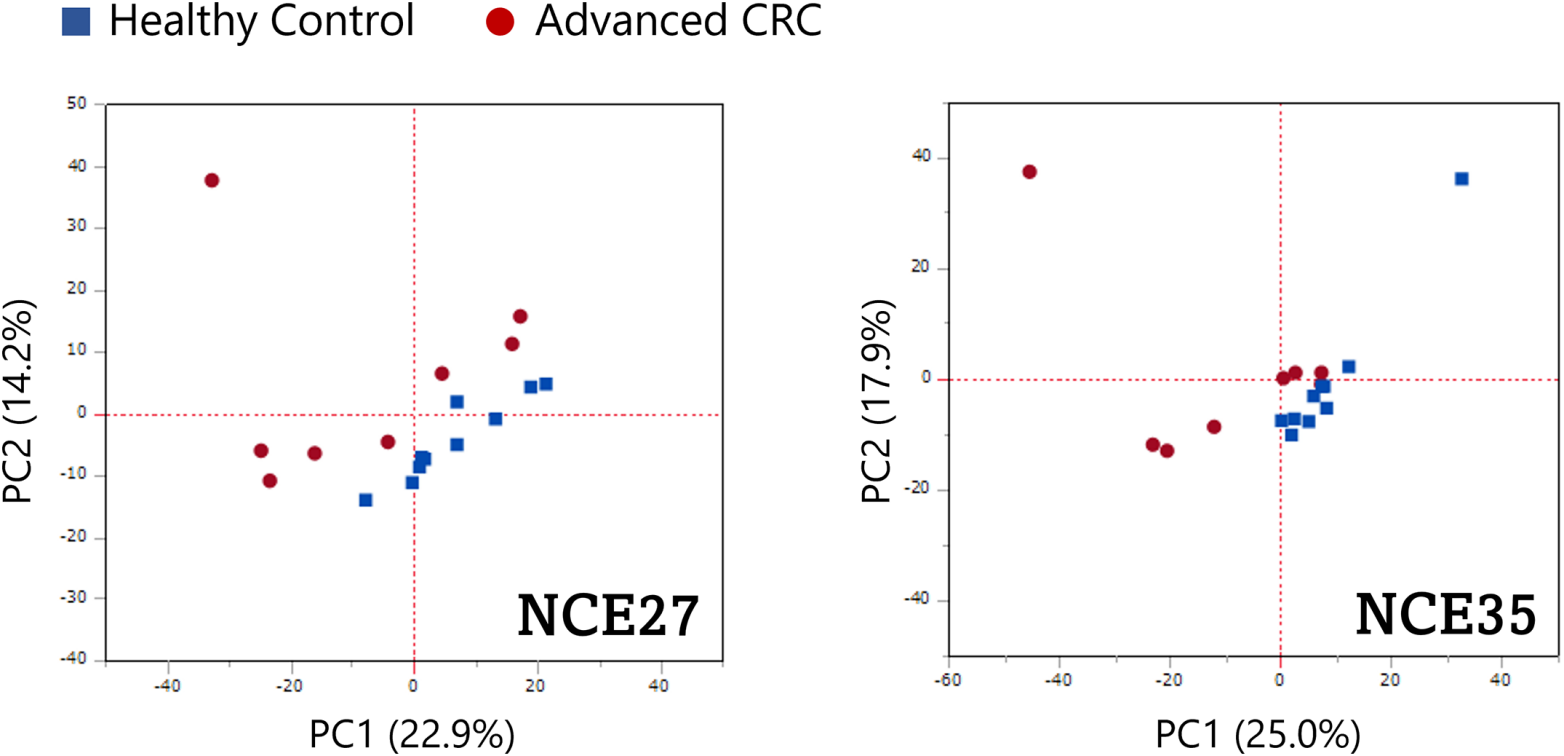
Potential ability of O-glycoproteins to distinguish patients with advanced CRC from healthy individuals. Principal component analysis (PCA) was performed on the peak area of the O-glycoforms.

To verify the diagnostic ability of O-glycoprotein to distinguish between patients with advanced CRC and normal individuals, the PCA algorithm was implemented on the peak area values traced for the precursor ions of O-glycoforms (**Figure 3**). Scatter diagrams show that patients with advanced CRC, and normal individuals have separate data distributions, suggesting that PCA revealed significant differences between the O-glycoform spectra in the serum of patients with advanced CRC and controls. Furthermore, normal specimens showed a more concentrated local distribution, whereas the distribution of cancer serum specimens appeared to be more widespread.

**Figure 4:**
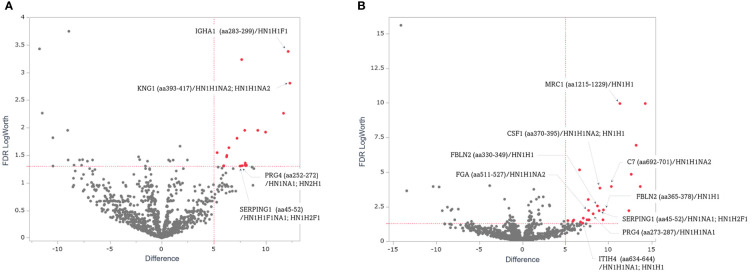
Quantitative comparison of the O-glycoforms of advanced CRC and healthy control serum samples. Volcano plots of O-glycoforms of patients with advanced CRC compared with those of the healthy controls. **(A)** NCE27, **(B)** NCE35. Red dots: difference ≥5.00; FDR LogWorth ≥1.3.

**Figure 5:**
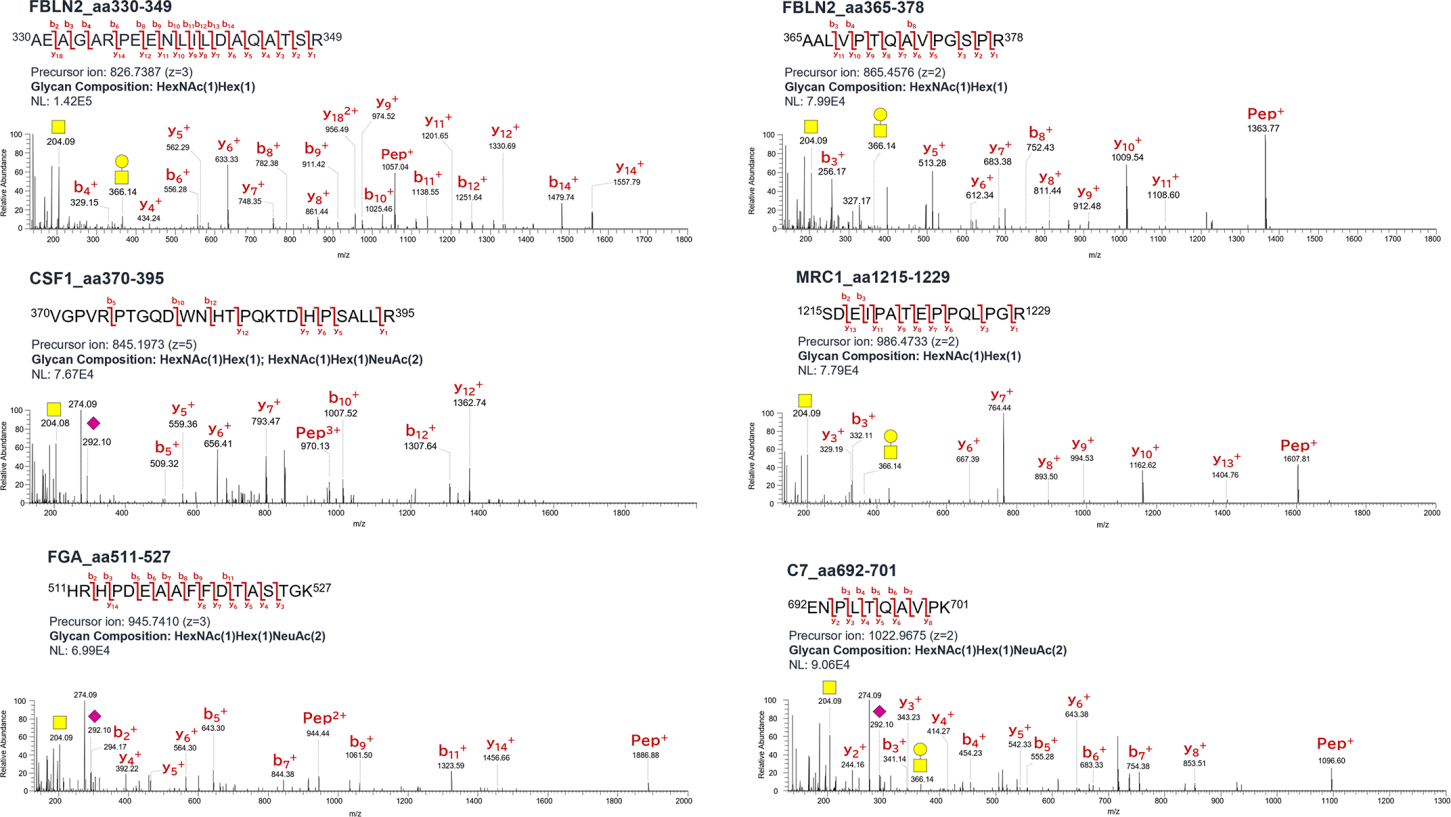
Product ion spectra of O-glycopeptides. Symbols: yellow circle, galactose; yellow square, *N*-acetylgalactosamine; purple diamond, *N*-acetylneuraminic acid; Hex, hexose; HexNAc, *N*-acetylhexosamine; NeuAc, *N*-acetylneuraminic acid.

Volcano plots revealed a comparative distribution of the peak area values traced for the precursor ions of the O-glycoforms between the two groups (**Figure 4**). O-glycoforms with statistically significant differences (≥5-Difference, FDR Logworth ≥1.3) are indicated by red circles. This suggests that in these 44 O-glycoforms, the O-glycan moieties were over-modified in a region-specific manner (**Supplemental Table 1**). The acquisition of reliable product ion spectra is essential for the selection of aberrant O-glycoproteins as marker candidates. Thus, we filtered O-glycoforms identified with byonic scores >400. Byonic score reflects the quality of the peptide-spectrum match, with scores above 400 indicating very high confidence in the identification (39). To maintain the validity of the independent data obtained on NCE27 and NCE35, we did not combine these mass spectrometry data in this study. Some conflicting results of fold change observed for the two fragmentation methods were resolved by selecting O-glycoforms with the higher Byonic score indicating confidence of identification. Furthermore, to construct a sandwich enzyme-linked immunosorbent assay (ELISA) system using an anti-protein antibody and a lectin, it is important to select aberrant O-glycoproteins that can efficiently bind to a specific lectin. Thus, highly O-glycosylated proteins, such as proteoglycan 4, kininogen-1, immunoglobulin heavy constant alpha 1, and plasma protease C1 inhibitor should not be selected because glycan heterogeneity is different for each modification site. We selected five glycoproteins: fibulin-2 (FBLN2), macrophage colony-stimulating factor 1 (CSF1), macrophage mannose receptor 1 (MRC1), fibrinogen alpha chain (FGA), and complement component C7 (C7), to assess the potential utility of O-glycoprotein markers in supporting the diagnosis and selection of treatment options for patients with advanced CRC. According to the Byonic search results, the T antigen: HexNAc (1)Hex(1) was found on 330–349 and 365–378 amino acid regions of FBLN2, respectively. In CSF1, T antigen: HexNAc(1)Hex(1) and di-ST: HexNAc(1)Hex(1)NeuAc(2), were found in the amino acid region of 370–395. T antigen: HexNAc(1)Hex(1) was found in 1215–1229 amino acid region of MRC1. di-ST was found on the 511–527 amino acid region of FGA and the 692–701 region of C7, respectively. All the O-glycoforms identified by the Byonic search engine were strongly supported by the product ion spectra (**Figure 5**). The typical oxonium ions are annotated on spectra including *m/z* at 204.08 (HexNAc), 274.09 and 292.10 (NeuAc), and 366.14 (HexNAc + Hex). The ions corresponding to peptide and peptide-related fragment that were correctly interpreted, have a high share of the total intensity in spectrum.

**Figure 6:**
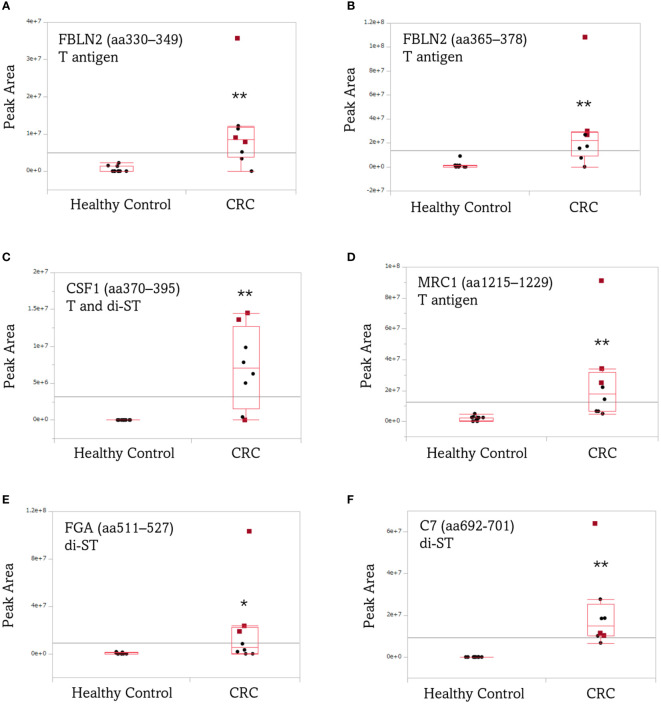
Box plots comparing the average peak area of aberrant O-glycoforms in the serum of patients with advanced CRC and healthy individuals. **(A)** FBLN2 (aa330–349)/T, **(B)** FBLN2 (aa365–378)/T, **(C)** CSF1 (aa370–395)/(T + di-ST), **(D)** MRC1 (aa1215–1229)/T, **(E)** FGA (aa511–527)/di-ST, **(F)** C7 (aa692-701)/di-ST. Each dot represents the O-glycoform level of an individual specimen. The line inside each box indicates the median and the lower and upper hinges correspond to the first and third quartiles. Wilcoxon matched-pairs signed rank test was performed using the peak area of the O-glycoform. Red squares: CEA/CA19-9 (+/+). **, *p* < 0.01; *, *p* < 0.05. CEA/CA19-9, carcinoembryonic antigen/carbohydrate antigen 19-9; FBLN2, fibulin-2; CSF1, colony-stimulating factor 1; MRC1, mannose receptor 1; FGA, fibrinogen alpha chain; C7, complement component C7.

**Figure 6** illustrates box plots comparing the average peak area values traced for the precursor ions of O-glycoform, upregulated in remarkably significant manner in the serum of patients with advanced CRC compared with the control. The average peak area values of FBLN2 (aa330–349 and aa365–378)/T, CSF1 (aa370–395)/(T + di-ST), MRC1 (aa1215–1229)/T, and C7 (aa692–701)/di-ST in the serum of patients with advanced CRC were significantly increased (*p* < 0.01). The increased serum level of FBLN2 (aa365–378)/T, MRC1 (aa1215–1229)/T, and FGA (aa511–527)/di-ST was commonly observed in CEA and CA19-9 positive patients (**Figure 6B, D, E**). In addition, it is worth noting that high peak area values were detected even in specimens that were negative for CEA and CA19-9, which are markers for advanced CRC (**Figure 6A, C, F**).

**Figure 7:**
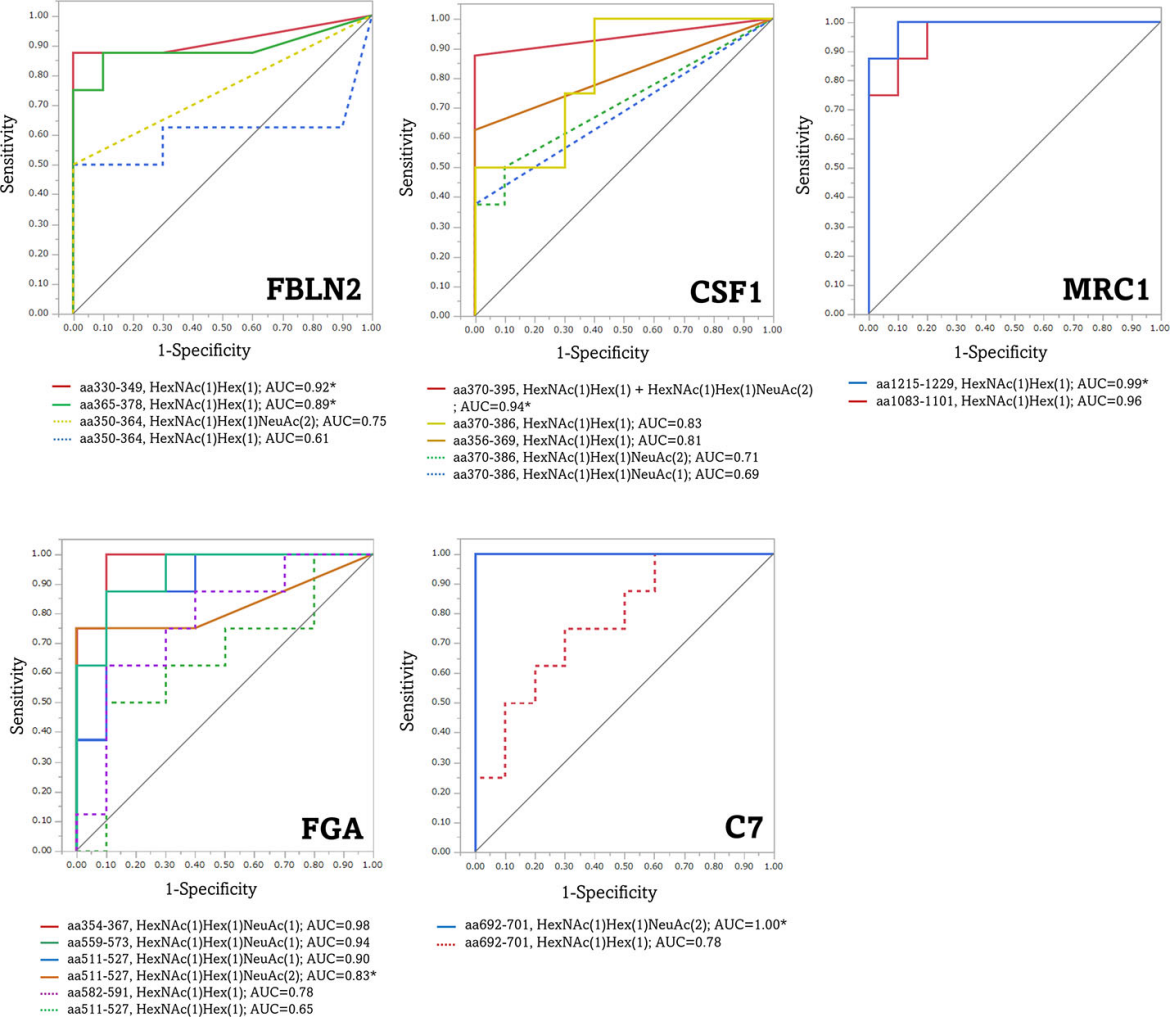
Receiver operating characteristic (ROC) curves for the diagnosis of advanced CRC by aberrant O-glycoforms. *, significant O-glycoforms. FBLN2, fibulin-2; CSF1, colony-stimulating factor 1; MRC1, macrophage mannose receptor 1; FGA, fibrinogen alpha chain; C7, complement component C7.

ROC analysis was performed to assess the diagnostic ability, to distinguish between patients with advanced CRC and healthy individuals, using the average peak area value traced for the precursor ion of O-glycoform derived from the five aberrant O-glycoproteins in serum with advanced CRC. As shown in **Figure 7**, the FBLN2 (aa330–349)/T antigen, CSF1 (aa370–395)/(T + di-ST), MRC1 (aa1083–1101 and aa1215–1229)/T antigen, FGA (aa354–367, aa511–527 and aa559–573)/ST or Sialyl Core 1, and C7 (aa692–701)/di-ST achieved an AUC value of >0.90, demonstrating that these O-glycoforms have high sensitivity and specificity for advanced CRC diagnosis. Of note, each O-glycopeptide of FBLN2 exhibited different AUC values. The greatest improvement in AUC values, compared to aa350-364/T, was noted for aa330-349/T (AUC 0.92 vs 0.61). In addition, it is worth noting that each O-glycoform showed different AUC values despite O-glycosylation to the same peptide region.

## 4 Discussion

This is the first study employing O-glycoproteomics to search for diagnostic markers for advanced CRC in clinical samples. We focused our screening targets, from patient sera, on proteins with cancer-related O-glycans, such as Tn, STn, T, ST, and di-ST antigens. We sequentially enriched O-glycosylated proteins using three different lectins that have an affinity for O-glycans. It is worth mentioning that the lectins used in this enrichment process are directly applicable to the subsequent sandwich ELISA using anti-protein antibodies and lectins. The reactivity between these lectins and glycopeptides seems to be less robust than that between lectins and glycoproteins. In our preliminary studies, the number of O-glycoforms identified is greatly reduced. Furthermore, the acetone-based glycopeptide enrichment method played an important role in compensating for the limitations of the GlycOCATCH affinity resin for sialoglycopeptides. Our O-glycoproteomics workflow may also enrich some N-glycopeptides. However, the identification is limited to glycopeptides with O-glycans entered in our database. The presence of some N-glycopeptides is unlikely to be an obstacle in our goal of identifying cancer-related O-glycopeptides. Indeed, the O-glycoproteomics platform used in this study enabled us to monitor the dynamic changes in over 2000 O-glycoforms in human serum samples. Appropriate NCE values for ideal fragmentation depend on glycopeptide size, amino acid composition, and glycan composition. In addition, fragmentation of glycopeptides is expected to require higher NCE values compared to peptides. We used two different NCEs to search for CRC-related O-glycoforms, because the acquisition of reliable product ion spectra is critical for the identification of many O-glycoforms. As shown in **Figure 2A**, changing the NCE value allows identification of different O-glycoforms.

Glycoforms modified by the six major types of O-glycans were extracted from all the O-glycoforms identified by the Byonic search engine, and compared between advanced CRC and normal sera. We set the maximum number of modifications of O-glycosylation sites per peptide as 2. Higher energy collision-induced dissociation fragmentation of O-glycopeptide cannot identify O-glycosylation sites when there are more than one glycosites. In such cases, we estimated the number of O-glycans from the glycan composition. As shown in **Figure 2C**, the major O-glycans with a high frequency of occurrence, both in serum with cancer, and in normal tissues were T, ST, and di-ST antigens. This result is consistent with previous reports that, O-glycans released in healthy human serum are composed of ST antigen, di-ST, and T antigen (40). Twenty polypeptide *N*-acetylgalactosamine transferases (ppGalNAcTs) are responsible for initiating mucin-type O-glycosylation, and differ with respect to their expression patterns and substrate selectivity in cells and tissues. In CRC tissues, the expression of polypeptide *N*-acetylgalactosaminyltransferase 6 (GALNT6) is particularly upregulated, and is involved in cell proliferation and adhesion (41). As shown in **Figure 3**, PCA using the peak area values of the identified O-glycoforms yielded separate clusters for advanced CRC patients and healthy individuals. The differences in typical O-glycoforms recognized between the two serum groups, suggest that the development of cancer-specific O-glycoproteins is due to aberrant substrate selection by ppGalNAcTs in patients with advanced CRC. This fact sheds light on O-glycoproteins as new clinical test indicators for advanced CRC.

We showed that O-glycopeptides from five proteins, FBLN2, CSF1, MRC1, FGA, and C7 were significantly elevated in the sera of patients with recurrent and advanced CRC. Product ion spectra of these O-glycopeptides were strongly supported with manual annotation. These O-glycoproteins may not necessarily be independent of cancer progression or malignancy. Basement membrane destabilization associated with the loss of FBLN2 contributes to increased cancer cell migration and invasion (42–44), and high expression of CSF1 and macrophage infiltration are associated with the tumor, node and metastasis (TNM) stage of CRC (45). Tumor-associated macrophages express high levels of MRC1, are considered to contribute to cancer progression, and are closely associated with poor cancer prognosis (46). Moreover, increased fibrinogen α-chain levels in the serum correlate with the hypercoagulable state in cancer patients (47). C7, one of the terminal complement proteins involved in the formation of the membrane-attack complex (48), is proposed as a prognostic marker for prostate cancer (49). The peak area values of the O-glycoform derived from these five glycoproteins were significantly increased in advanced CRC specimens as compared to the normal specimens (**Figure 6**). It is noteworthy that the values were relatively high, even in advanced CRC specimens, in which both the existing markers, CEA and CA19-9 were negative. This indicates that a close coordination of these marker candidate proteins with CEA and CA19-9 may enable more accurate monitoring of the patient’s condition. This suggestion is also supported by **Figure 7**, which shows that the AUC values of the aberrant O-glycoforms are approximately ≥0.8. As mentioned above, when detecting advanced CRC in combination with an anti-protein antibody such as sandwich ELISA, MPL, and jacalin or SNL should be effective in detecting these O-glycoproteins. Our results confirm the reactivity with these lectins during the enrichment of O-glycoproteins from the serum. The results of O-glycopeptides do not guarantee the results of ELISA for glycoproteins. This is because we cannot deny the existence of an O-glycosylation region that we have not identified. It is necessary to analyze candidate marker proteins in detail after enrichment by immunoprecipitation. Detailed glycopeptide mapping should be performed prior to ELISA.

In conclusion, we have demonstrated aberrant O-glycosylation in advanced CRC using a novel O-glycoproteomics platform. O-glycosylation differed between patients with advanced CRC and healthy individuals, suggesting the possibility that they can be used for advanced CRC diagnosis. Generation of specific antibody against the aberrant O-glycoforms is challenging. Further validation with multiple specimens, and in-depth site-specific O-glycosylation analysis may pave way for the development of novel diagnostics for advanced CRC targeting O-glycoproteins that can support an improved quality of life in patients.

## Data availability statement

The datasets presented in this study can be found in online repositories. The names of the repository/repositories and accession number(s) can be found in the article/**Supplementary Material**.

## Ethics statement

The studies involving human participants were reviewed and approved by Institutional Ethics Committee of Yokohama City University. The patients/participants provided their written informed consent to participate in this study.

## Author contributions

DT, YI, and NKa conceptualized the study and wrote the draft manuscript. DT and SO performed experiments and data analysis. NKo, MT, and YI provided intellectual support and expertise in oncology. All authors contributed to the article and approved the submitted version.

## References

[1] Siegel R Naishadham D Jemal A. Cancer statistics, 2013. CA Cancer J Clin (2013) 63(1):11–30. doi: 10.3322/caac.21166 23335087

[2] Guinney J Dienstmann R Wang X de Reyniès A Schlicker A Soneson C. The consensus molecular subtypes of colorectal cancer. Nat Med (2015) 21(11):1350–56. doi: 10.1038/nm.3967 PMC4636487 26457759

[3] Issa IA Noureddine M. Colorectal cancer screening: An updated review of the available options. World J Gastroenterol (2017) 23(28):5086–96. doi: 10.3748/wjg.v23.i28.5086 PMC5537177 28811705

[4] DeStefanis RA Kratz JD Emmerich PB Deming DA. Targeted therapy in metastatic colorectal cancer: Current standards and novel agents in review. Curr Colorectal Cancer Rep (2019) 15(2):61–9. doi: 10.1007/s11888-019-00430-6 PMC6528813 31130830

[5] Martinez-Romero J Bueno-Fortes S Martín-Merino M Ramirez de Molina A De Las Rivas J. Survival marker genes of colorectal cancer derived from consistent transcriptomic profiling. BMC Genomics (2018) 19(Suppl 8):857. doi: 10.1186/s12864-018-5193-9 30537927 PMC6288855

[6] Gharib E Nasrinasrabadi P Zali MR. Development and validation of a lipogenic genes panel for diagnosis and recurrence of colorectal cancer. PloS One (2020) 15(3):e0229864. doi: 10.1371/journal.pone.0229864 32155177 PMC7064220

[7] Chen F Dai X Zhou CC Li KX Zhang YJ Lou XY. Integrated analysis of the faecal metagenome and serum metabolome reveals the role of gut microbiome-associated metabolites in the detection of colorectal cancer and adenoma. Gut (2022) 71(7):1315–25. doi: 10.1136/gutjnl-2020-323476 PMC9185821 34462336

[8] Farshidfar F Kopciuk KA Hilsden R McGregor SE Mazurak VC Buie WD. A quantitative multimodal metabolomic assay for colorectal cancer. BMC Cancer (2018) 18(1):26. doi: 10.1186/s12885-017-3923-z 29301511 PMC5755335

[9] Harlid S Harbs J Myte R Brunius C Gunter MJ Palmqvist R. A two-tiered targeted proteomics approach to identify pre-diagnostic biomarkers of colorectal cancer risk. Sci Rep (2021) 11(1):5151. doi: 10.1038/s41598-021-83968-6 33664295 PMC7933352

[10] Bhardwaj M Weigl K Tikk K Holland-Letz T Schrotz-King P Borchers CH. Multiplex quantitation of 270 plasma protein markers to identify a signature for early detection of colorectal cancer. Eur J Cancer (2020) 127:30–40. doi: 10.1016/j.ejca.2019.11.021 31972396

[11] Apweiler R Hermjakob H Sharon N. On the frequency of protein glycosylation, as deduced from analysis of the SWISS-PROT database. Biochim Biophys Acta (1999) 1473(1):4–8. doi: 10.1016/s0304-4165(99)00165-8 10580125

[12] Reily C Stewart TJ Renfrow MB Novak J. Glycosylation in health and disease. Nat Rev Nephrol (2019) 15(6):346–66. doi: 10.1038/s41581-019-0129-4 PMC6590709 30858582

[13] Raman J Guan Y Perrine CL Gerken TA Tabak LA. UDP-N-acetyl-α-D-galactosamine: Polypeptide *N*-acetylgalactosaminyltransferases: completion of the family tree. Glycobiology (2012) 22(6):768–77. doi: 10.1093/glycob/cwr183 PMC3336867 22186971

[14] Steentoft C Vakhrushev SY Joshi HJ Kong Y Vester-Christensen MB Schjoldager KT. Precision mapping of the human O-GalNAc glycoproteome through SimpleCell technology. EMBO J (2013) 32(10):1478–88. doi: 10.1038/emboj.2013.79 PMC3655468 23584533

[15] Hintze J Ye Z Narimatsu Y Madsen TD Joshi HJ Goth CK. Probing the contribution of individual polypeptide GalNAc-transferase isoforms to the O-glycoproteome by inducible expression in isogenic cell lines. J Biol Chem (2018) 293(49):19064–77. doi: 10.1074/jbc.RA118.004516 PMC6295722 30327431

[16] Bennett EP Mandel U Clausen H Gerken TA Fritz TA Tabak LA. Control of mucin-type O-glycosylation: A classification of the polypeptide GalNAc-transferase gene family. Glycobiology (2012) 22(6):736–56. doi: 10.1093/glycob/cwr182 PMC3409716 22183981

[17] Acharya A Markar SR Matar M Ni M Hanna GB. Use of tumor markers in gastrointestinal cancers: Surgeon perceptions and cost-benefit trade-off analysis. Ann Surg Oncol (2017) 24(5):1165–73. doi: 10.1245/s10434-016-5717-y PMC5374165 28008574

[18] Thomas D Rathinavel AK Radhakrishnan P. Altered glycosylation in cancer. a promising target for biomarkers and therapeutics. Biochim Biophys Acta Rev Cancer (2021) 1875(1):188464. doi: 10.1016/j.bbcan.2020.188464 33157161 PMC7855613

[19] Aoyagi Y Suzuki Y Isemura M Nomoto M Sekine C Igarashi K. The fucosylation index of alpha-fetoprotein and its usefulness in the early diagnosis of hepatocellular carcinoma. Cancer (1988) 61(4):769–74. doi: 10.1002/1097-0142(19880215)61:4<769::aid-cncr2820610422>3.0.co;2-m 2448024

[20] Katoh H Nakamura K Tanaka T Satomura S Matsuura S. Automatic and simultaneous analysis of lens culinaris agglutinin-reactive alpha-fetoprotein ratio and total alpha-fetoprotein concentration. Anal Chem (1998) 70(10):2110–14. doi: 10.1021/ac971280c 9608849

[21] Kagebayashi C Yamaguchi I Akinaga A Kitano H Yokoyama K Satomura M. Automated immunoassay system for AFP-L3% using on-chip electrokinetic reaction and separation by affinity electrophoresis. Anal Biochem (2009) 388(2):306–11. doi: 10.1016/j.ab.2009.02.030 19250915

[22] Ito N Yamada M Morishita K Nojima S Motooka K Sakata N. Identification of fucosylated haptoglobin-producing cells in pancreatic cancer tissue and its molecular mechanism. Glycoconj J (2021) 38(1):45–54. doi: 10.1007/s10719-020-09970-8 33523362

[23] Morishita K Ito N Koda S Maeda M Nakayama K Yoshida K. Haptoglobin phenotype is a critical factor in the use of fucosylated haptoglobin for pancreatic cancer diagnosis. Clin Chim Acta (2018) 487:84–9. doi: 10.1016/j.cca.2018.09.001 30189188

[24] Springer GF Taylor CR Howard DR Tegtmeyer H Desai PR Murthy SM. TN, a carcinoma-associated antigen, reacts with anti-TN of normal human sera. Cancer (1985) 55(3):561–69. doi: 10.1002/1097-0142(19850201)55:3<561::aid-cncr2820550315>3.0.co;2-1 2981150

[25] Fu C Zhao H Wang Y Cai H Xiao Y Zeng Y. Tumor-associated antigens: TN antigen, sTn antigen, and T antigen. HLA (2016) 88(6):275–86. doi: 10.1111/tan.12900 27679419

[26] Lugo R Ávila-Nava A García-Pérez R Herrera-Escalante S de la Cruz-Acosta J Gutiérrez-Solis AL. Systematic review and meta-analysis of the clinical survival significance of sialyl-tn expression in histological tissues from cancer patients. Transl Cancer Res (2020) 9(2):547–55. doi: 10.21037/tcr.2019.11.53 PMC8799194 35117399

[27] Munkley J. The role of sialyl-tn in cancer. Int J Mol Sci (2016) 17(3):275. doi: 10.3390/ijms17030275 26927062 PMC4813139

[28] Peixoto A Fernandes E Gaiteiro C Lima L Azevedo R Soares J. Hypoxia enhances the malignant nature of bladder cancer cells and concomitantly antagonizes protein O-glycosylation extension. Oncotarget (2016) 7(39):63138–157. doi: 10.18632/oncotarget.11257 PMC5325352 27542232

[29] Chia J Goh G Bard F. Short O-GalNAc glycans: Regulation and role in tumor development and clinical perspectives. Biochim Biophys Acta (2016) 1860(8):1623–39. doi: 10.1016/j.bbagen.2016.03.008 26968459

[30] Jiang Y Liu Z Xu F Dong X Cheng Y Hu Y. Aberrant O-glycosylation contributes to tumorigenesis in human colorectal cancer. J Cell Mol Med (2018) 22(10):4875–85. doi: 10.1111/jcmm.13752 PMC6156240 29999571

[31] Wandall HH Nielsen MAI King-Smith S de Haan N Bagdonaite I. Global functions of O-glycosylation: Promises and challenges in O-glycobiology. FEBS J (2021) 288(24):7183–212. doi: 10.1111/febs.16148 34346177

[32] Chen R Seebun D Ye M Zou H Figeys D. Site-specific characterization of cell membrane n-glycosylation with integrated hydrophilic interaction chromatography solid phase extraction and LC-MS/MS. J Proteomics (2014) 103:194–203. doi: 10.1016/j.jprot.2014.03.040 24721674

[33] Qu Y Sun L Zhang Z Dovichi NJ. Site-specific glycan heterogeneity characterization by hydrophilic interaction liquid chromatography solid-phase extraction, reversed-phase liquid chromatography fractionation, and capillary zone electrophoresis-electrospray ionization-tandem mass spectrometry. Anal Chem (2018) 90(2):1223–33. doi: 10.1021/acs.analchem.7b03912 PMC5771954 29231704

[34] Togayachi A Tomioka A Fujita M Sukegawa M Noro E Takakura D. Identification of poly-n-acetyllactosamine-carrying glycoproteins from HL-60 human promyelocytic leukemia cells using a site-specific glycome analysis method, glyco-RIDGE. J Am Soc Mass Spectrom (2018) 29(6):1138–52. doi: 10.1007/s13361-018-1938-6 PMC6004004 29675740

[35] Takakura D Harazono A Hashii N Kawasaki N. Selective glycopeptide profiling by acetone enrichment and LC/MS. J Proteomics (2014) 101:17–30. doi: 10.1016/j.jprot.2014.02.005 24530628

[36] Mancera-Arteu M Giménez E Benavente F Barbosa J Sanz-Nebot V. Analysis of O-glycopeptides by acetone enrichment and capillary electrophoresis-mass spectrometry. J Proteome Res (2017) 16(11):4166–76. doi: 10.1021/acs.jproteome.7b00524 28944674

[37] Turiák L Sugár S Ács A Tóth G Gömöry Á Telekes A. Site-specific n-glycosylation of HeLa cell glycoproteins. Sci Rep (2019) 9(1):14822. doi: 10.1038/s41598-019-51428-x 31616032 PMC6794373

[38] Trastoy B Naegeli A Anso I Sjögren J Guerin ME. Structural basis of mammalian mucin processing by the human gut O-glycopeptidase OgpA from akkermansia muciniphila. Nat Commun (2020) 11(1):4844. doi: 10.1038/s41467-020-18696-y 32973204 PMC7518263

[39] Bern M Kil YJ Becker C. Byonic: Advanced peptide and protein identification software. Curr Protoc Bioinf (2012) Chapter 13:Unit13.20. doi: 10.1002/0471250953.bi1320s40 PMC3545648 23255153

[40] Yabu M Korekane H Miyamoto Y. Precise structural analysis of O-linked oligosaccharides in human serum. Glycobiology (2014) 24(6):542–53. doi: 10.1093/glycob/cwu022 24663386

[41] Lavrsen K Dabelsteen S Vakhrushev SY Levann AMR Haue AD Dylander A. *De novo* expression of human polypeptide *N*-acetylgalactosaminyltransferase 6 (GalNAc-T6) in colon adenocarcinoma inhibits the differentiation of colonic epithelium. J Biol Chem (2018) 293(4):1298–314. doi: 10.1074/jbc.M117.812826 PMC5787806 29187600

[42] Yi CH Smith DJ West WW Hollingsworth MA. Loss of fibulin-2 expression is associated with breast cancer progression. Am J Pathol (2007) 170(5):1535–45. doi: 10.2353/ajpath.2007.060478 PMC1854949 17456760

[43] Senapati S Gnanapragassam VS Moniaux N Momi N Batra SK. Role of MUC4-NIDO domain in the MUC4-mediated metastasis of pancreatic cancer cells. Oncogene (2012) 31(28):3346–56. doi: 10.1038/onc.2011.505 PMC3298579 22105367

[44] Ibrahim AM Sabet S El-Ghor AA Kamel N Anis SE Morris JS. Fibulin-2 is required for basement membrane integrity of mammary epithelium. Sci Rep (2018) 8(1):14139. doi: 10.1038/s41598-018-32507-x 30237579 PMC6148073

[45] Wang H Shao Q Sun J Ma C Gao W Wang Q. Interactions between colon cancer cells and tumor-infiltrated macrophages depending on cancer cell-derived colony stimulating factor 1. Oncoimmunology (2016) 5(4):e1122157. doi: 10.1080/2162402X.2015.1122157 27141406 PMC4839327

[46] Fan W Yang X Huang F Tong X Zhu L Wang S. Identification of CD206 as a potential biomarker of cancer stem-like cells and therapeutic agent in liver cancer. Oncol Lett (2019) 18(3):3218–26. doi: 10.3892/ol.2019.10673 PMC6704291 31452799

[47] Lv P Liu Z Xu B Tang C Li X Qin H. Exploratory study on application of MALDI-TOF-MS to detect serum and urine peptides related to small cell lung carcinoma. Mol Med Rep (2020) 21(1):51–60. doi: 10.3892/mmr.2019.10794 31746355 PMC6896340

[48] Ain D Shaikh T Manimala S Ghebrehiwet B. The role of complement in the tumor microenvironment. FAC Rev (2021) 10:80. doi: 10.12703/r/10-80 35028645 PMC8725651

[49] Chen Z Yan X Du GW Tuoheti K Bai XJ Wu HH. Complement C7 (C7), a potential tumor suppressor, is an immune-related prognostic biomarker in prostate cancer (PC). Front Oncol (2020) 10:1532. doi: 10.3389/fonc.2020.01532 32984006 PMC7477933

